# Tumor risks of finerenone in patients with type 2 diabetes mellitus complicated with chronic kidney disease: a meta-analysis and systematic review of randomized controlled trials

**DOI:** 10.3389/fphar.2023.1237583

**Published:** 2024-01-11

**Authors:** Yue Du, Gui Cao, Linlin Gu, Yuehong Chen, Jingyu Liu

**Affiliations:** ^1^ Department of Endocrinology, Chengdu Seventh People’s Hospital (Affiliated Cancer Hospital of Chengdu Medical College), Chengdu, China; ^2^ Department of Rheumatology and Immunology, West China Hospital, Sichuan University, Chengdu, China

**Keywords:** finerenone, T2DM, chronic kidney disease, tumor, meta-analysis

## Abstract

**Introduction:** This study aimed to assess the tumor risk of finerenone in individuals with type 2 diabetes mellitus (T2DM) aggravated by chronic kidney disease (CKD).

**Methods:** A thorough search in the OVID Medline, OVID EMBASE, and Cochrane Library databases from their creation through 2 November 2022 yielded randomized controlled trials (RCTs) reporting on the tumor risks of finerenone in patients with T2DM complicated with CKD. A pair of reviewers selected the relevant studies based on selection criteria, collected data, and assessed the methodological quality of eligible RCTs. The Peto odds ratio (OR) with a 95% confidence interval (CI) was calculated, and subgroup analysis of tumor nature, tumor origin system, tumor origin organ, and follow-up time was performed. Furthermore, Egger’s test was implemented to determine publication bias.

**Results:** Four RCTs with 14,875 participants who had a low-to-moderate risk of bias were included. Compared with placebo treatment, finerenone did not increase the risk of overall neoplasms (Peto OR = 0.97; 95% CI, 0.83–1.14), malignant neoplasms (Peto OR = 1.03; 95% CI, 0.86–1.23), benign neoplasms (Peto OR = 0.94; 95% CI, 0.50–1.80), or *in situ* neoplasms (Peto OR = 0.14; 95% CI, 0.01–2.17). Subgroup analysis of the tumor origin system showed that finerenone was associated with an increased risk of malignant neoplasms of urinary tract compared with placebo treatment (Peto OR = 1.69; 95% CI, 1.07–2.67). The results were found to be robust in sensitivity analysis, and there was no indication of publication bias.

**Discussion:** Finerenone is not associated with an increased risk of overall tumors, but it may be linked to an increased risk of malignant neoplasms in urinary tract. Additional well-planned cohort studies in larger research populations are needed to corroborate these findings.

**Systematic Review Registration:** https://www.crd.york.ac.uk/prospero/display_record.php?ID=CRD42022374101, Identifier CRD42022374101.

## 1 Introduction

Diabetes mellitus is a major chronic disease worldwide (Author Anyonomus, 2009). According to the latest data from the International Diabetes Federation, in 2021, 537 million adults worldwide aged 20–79 years have diabetes (i.e., approximately one in ten of the global population), and this number is expected to increase to reach 643 million by 2035 and 783 million by 2045 (https://diabetesatlas.org/). Diabetes is characterized by high blood glucose levels that can lead to microvascular and macrovascular diseases, which are the main causes of chronic kidney disease (CKD) (Hardin et al., 1956; Stratton et al., 2000; Sarwar et al., 2010). More importantly, type 2 diabetes mellitus (T2DM) is reported to be associated with an increased risk of cancer (Shlomai et al., 2016), which may be caused by hyperglycemia, hyperinsulinemia, and obesity. Chronically elevated endogenous insulin and/or IGF-1 levels can increase mitogenic signaling and promote tumor growth and metastasis (Renehan et al., 2008; Shlomai et al., 2016), placing a huge economic burden on the patient, their family, and society.

Significant increases in site-specific cancer risk in patients with T2DM have been reported, the most notable of which are risks of breast cancer [risk ratio (RR) = 1.20; 95% confidence interval (CI), 1.12–1.28], intrahepatic bile duct cancer (RR = 1.20; 95% CI, 1.57–2.46), colorectal cancer (RR = 1.27; 95% CI, 1.21–1.34), endometrial cancer (RR = 1.97; 95% CI, 1.71–2.27), hepatocellular carcinoma (RR = 2.43; 95% CI, 1.67–3.35), gallbladder cancer (RR = 1.73; 95% CI, 1.40–2.14), and pancreatic cancer (RR = 1.94; 95% CI, 1.66–2.27), except for the risk of localized prostate cancer (RR = 0.80; 95% CI, 0.70–0.90), which demonstrated the opposite effect (Tsilidis et al., 2015; Pearson-Stuttard et al., 2021). A 10-year prospective cohort study from Korea in 2005 showed that the incidence of cancer increased with blood glucose levels, with the highest corresponding increase being for pancreatic cancer in men [hazard ratio (HR) = 2.09; 95% CI, 1.70–2.58] and cervical cancer in women (HR = 2.20; 95% CI, 1.90–2.54). Fasting plasma glucose (FBG) ≥7.8 mmol/L was associated with higher mortality for all cancers (men: HR = 1.29; 95% CI, 1.22–1.37; women: HR = 1.23; 95% CI, 1.09–1.39) (Jee et al., 2005). Moreover, a duration of diabetes of more than 5 years and FBG ≥10.0 mmol/L were associated with increased cancer risks (HR = 2.35; 95% CI, 1.77–3.13) compared to those with an FBG <6.0 mmol/L (Shen et al., 2023).

Finerenone, a new type of nonsteroidal mineralocorticoid receptor (MR) antagonist, is a naphthyridine derivative developed based on the dihydropyridine structure and has been identified through the high-throughput screening of millions of compounds (Bärfacker et al., 2012). In the human body, 90% of finerenone is metabolized by cytochrome P450 (CYP) 3A4 in the intestinal wall and liver, with the remaining 10% being metabolized by CYP2C8 (Heinig et al., 2016; Gerisch et al., 2018; Heinig et al., 2018). Around 80% of its metabolites are excreted in the urine, while the remainder are excreted in the feces. *In vivo* studies have shown that most metabolites of finerenone in human plasma are naphthyridine derivatives (48.9% for M1; 21.5% for M2; and 9.0% for M3), and M1, M2, and M3 were not found to have pharmacological activity on human mineralocorticoid receptors (Heinig et al., 2016; Gerisch et al., 2018). Gerisch et al. (2018) found that the excretion rates of M1 were <1.5%, which was negligible, and that elimination of M2 and M3 occurred mainly through the kidneys. Urinary excretion of M2 and M3 decreases with renal function (Heinig et al., 2016). Finerenone is evenly distributed in the heart and kidneys (Kolkhof et al., 2014) and has a stereoscopic structure and side chain; thus, it can bind to the MR more completely and has a stronger MR-antagonistic effect than spironolactone (SPIR) and eplerenone (Bärfacker et al., 2012; Jaisser and Farman, 2016). Additionally, because finerenone has a low affinity for androgen and progesterone receptors, it has no negative effects associated with sex hormones (Bärfacker et al., 2012; Jaisser and Farman, 2016). Finerenone has strong anti-inflammatory and anti-fibrotic effects, inhibiting the progression of CKD and reducing the risk of cardiovascular events (Bakris et al., 2020; Pitt et al., 2021). Finerenone was approved by the US Food and Drug Administration in 2021 for the treatment of adult patients with T2DM compounded by CKD to postpone the ongoing decline in the estimated glomerular filtration rate, and reduce the risk of cardiovascular events (Frampton, 2021). Both the American Heart Association and the American Diabetes Association approved finerenone in 2022 to reduce the risk of cardiovascular events and slow the progression of renal disease, respectively (Draznin et al., 2022; Joseph et al., 2022).

Considering that T2DM is associated with increased cancer risk, there is still no evidence to determine whether treatment with finerenone affects the development of cancer. Therefore, this systematic review and meta-analysis were performed to illustrate the tumor risks of finerenone in patients with T2DM complicated by CKD using currently available evidence from randomized controlled trials (RCTs).

## 2 Materials and methods

### 2.1 Setting

Meta-analysis and systematic review were conducted to assess the risk of tumor development in T2DM patients with CKD who were treated with finerenone (Moher et al., 2009). The study was registered on PROSPERO (CRD42022374101) and adhered to PRISMA guidelines for reporting.

### 2.2 Eligibility criteria

The trial comprised patients (P) with T2DM and CKD, and the intervention (I) was finerenone at any dose or usage. The comparison (C) group could receive any treatment except for finerenone. The outcome (O) of interest was the occurrence of any type of tumor, regardless of its nature. Only RCTs were considered for inclusion in the study (S), while duplicates, letters, abstracts, and studies with irrelevant results were excluded.

### 2.3 Search strategy

OVID Medline, OVID EMBASE, and Cochrane Library databases were searched for relevant studies from their inception to 2 November 2022, using both MeSH terms and keywords with no language restrictions. Among the search terms were “finerenone” and “RCT”. The search strategy is outlined in detail in the supplementary material. Additionally, we manually searched reference lists of included studies and clinicaltrials.gov for potentially eligible studies. Human RCTs were included, while non-randomized trials, studies without control or placebo groups, animal studies, and *in vitro* studies were excluded. The focus was on human participants.

### 2.4 Study selection

A team of reviewers (YD, GC, or LG) thoroughly examined the study selection, including preliminary screening of titles and abstracts as well as full-text reading. This was done in accordance with the study selection criteria, and any potentially relevant materials were identified through manual checks of reference lists and unpublished data from clinicaltrials.gov. Any disagreements that arose were resolved by a third reviewer (YC or JL).

### 2.5 Data extraction

Reviewers (YD, GC, or LG) gathered important information such as the registration number of the trial, date of publication or release, trial duration, number of tumors and participants, tumor nature, and location of origin. If there were any discrepancies, a third reviewer (YC or JL) was consulted to resolve the issue.

### 2.6 Methodological quality assessment

The Cochrane Collaboration tool was used to assess the risk of bias in the included RCTs (Higgins et al., 2011). Among the evaluation criteria were the generation of a random sequence, participant and personnel blinding, concealment of allocation, blinding of outcome assessment, insufficient outcome data, and selective reporting. To assess each item, a “yes” answer with a detailed description was considered low-risk, a “yes” answer without a detailed description was uncertain, and a “yes” answer with an inappropriate method or non-performance was considered high-risk. This overall evidence was used to determine the risk of bias in the studies. Two reviewers (YD and GC) assessed the risk of bias, and any discrepancies were resolved by a third reviewer (LG).

### 2.7 Data analyses

Data were combined with the RevMan software version 5.4. Considering the very few tumor events, the effect size was estimated using a Peto OR with a 95% CI (Bradburn et al., 2007; AHRQ Methods for Effective Health Care, 2008), because it performs well when dealing with sparse events (<1%) (Bradburn et al., 2007). I^2^ and heterogeneity *p*-values were applied to assess clinical diversity at a 0.1 level. The Cochrane Manual recommended that I2 values of 25%, 50%, and 75% indicate low, moderate, and high heterogeneity, respectively (Higgins et al., 2003). The subgroup analyses were carried out in accordance with tumor nature, tumor origin system, tumor origin organ, and follow-up duration, as specified beforehand. The robustness of the findings was examined using sensitivity analysis utilizing the Mantel-Haenszel random-effect model. To assess publication bias, Egger’s test was performed via STATA software, and *p* < 0.05 was considered significant. The International Classification of Diseases (ICD10) of the World Health Organization, 10th edition, was employed to classify tumor nature and originating system as follows: C00-C97 for malignant neoplasms, D10-D36 for benign neoplasms, D00-D009 for *in situ* neoplasms, and D37-D48 for neoplasms of uncertain or unknown behavior.

### 2.8 Patient and public involvement

There are no ethical concerns or patient involvement in this systematic review and meta-analysis.

## 3 Results

### 3.1 Study selection

**FIGURE 1:**
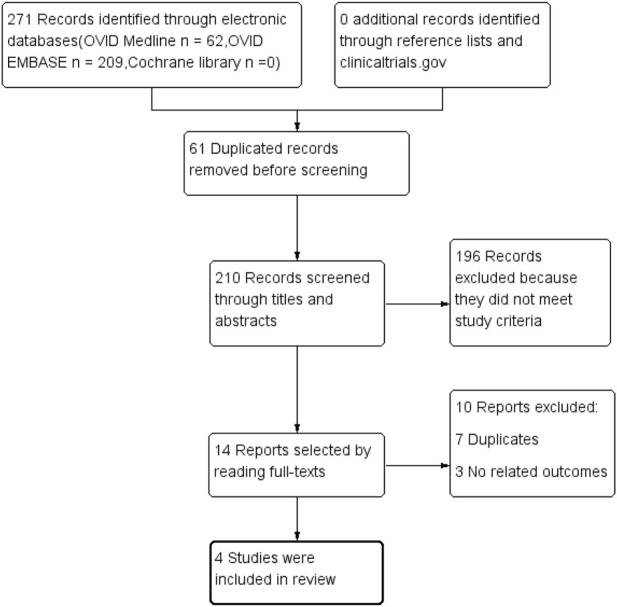
Study selection flowchart.

A total of 271 records were searched in electronic databases. After removing duplicates (*n* = 61), the title and abstract screening excluded unrelated records (*n* = 196). After reading the full texts, reports with no relevant outcomes (*n* = 3) or duplicate studies (*n* = 7) were excluded. Finally, four studies [NCT01807221 (Filippatos et al., 2016), NCT01874431 (Bakris et al., 2015), NCT02540993 (Bakris et al., 2020), and NCT02545049 (Pitt et al., 2021)] involving 14,875 patients with T2DM complicated with CKD were included (Figure 1). The manual review included no additional studies.

### 3.2 Characteristics of included studies

The included studies’ follow-up periods ranged from 3 to 40.8 months. The number of participants varied from 821 to 7,352. Approximately 70.75% of participants were men, and the mean patient age was 66.3 years, and the mean estimated glomerular filtration rate (eGFR, calculated with the use of the Chronic Kidney Disease Epidemiology Collaboration formula) of 58.2 mL per minute per 1.73 m^2^, and the most of CKD stages are 2–3. The common oral dosage of finerenone was 10 or 20 mg once daily (Table 1).

**TABLE 1:** Characteristics of included RCTs.

Included RCTs	Year of results first posted	Duration of follow-up (months)	No. of participants (female/male)	Age (years, mean ± SD)	eGFR (ml/min/1.73 m^2^, mean ±SD)	Treatments (no. of participants)
NCT01807221	2016	3	1,055	71.2 ± 10.1	53 ± 18	Finerenone 2.5–5 mg qd (172)
Filippatos et al. (2016)			(239/816)			Finerenone 5–10 mg qd (163)
						Finerenone 7.5–15 mg qd (167)
						Finerenone 10–20 mg qd (169)
						Finerenone 15–20 mg qd (163)
						Eplerenone (221)
NCT01874431	2015	3	821	64.2 ± 9.2	67.6 ± 21.7	Finerenone 1.25 mg qd (96)
Bakris et al. (2015)			(182/639)			Finerenone 2.5 mg qd (92)
						Finerenone 5 mg qd (100)
						Finerenone 7.5 mg qd (97)
						Finerenone 10 mg qd (98)
						Finerenone 15 mg qd (125)
						Finerenone 20 mg qd (119)
						Placebo (94)
NCT02540993	2020	31.2	5,674	65.6 ± 9.1	44.3 ± 12.6	Finerenone 10/20 mg qd (2,833)
Bakris et al. (2020)			(1,691/3,983)			Placebo (2,841)
NCT02545049	2021	40.8	7,352	64.1 ± 9.8	67.8 ± 21.7	Finerenone 10/20 mg qd (3,686)
Pitt et al. (2021)			(2,247/5,105)			Placebo (3,666)

qd = once a day, bid = twice a day.

### 3.3 Methodological quality

**FIGURE 2:**
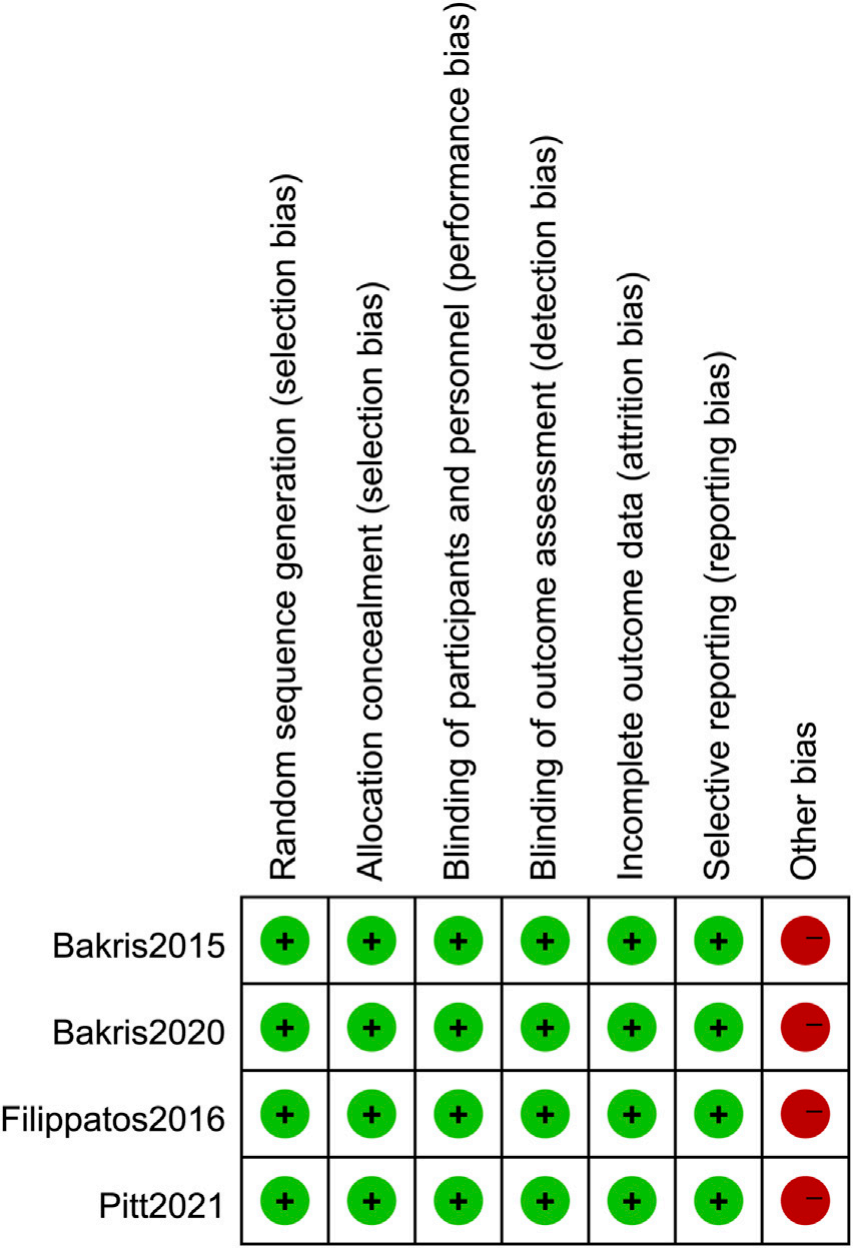
Risk of bias summary.

**FIGURE 3:**
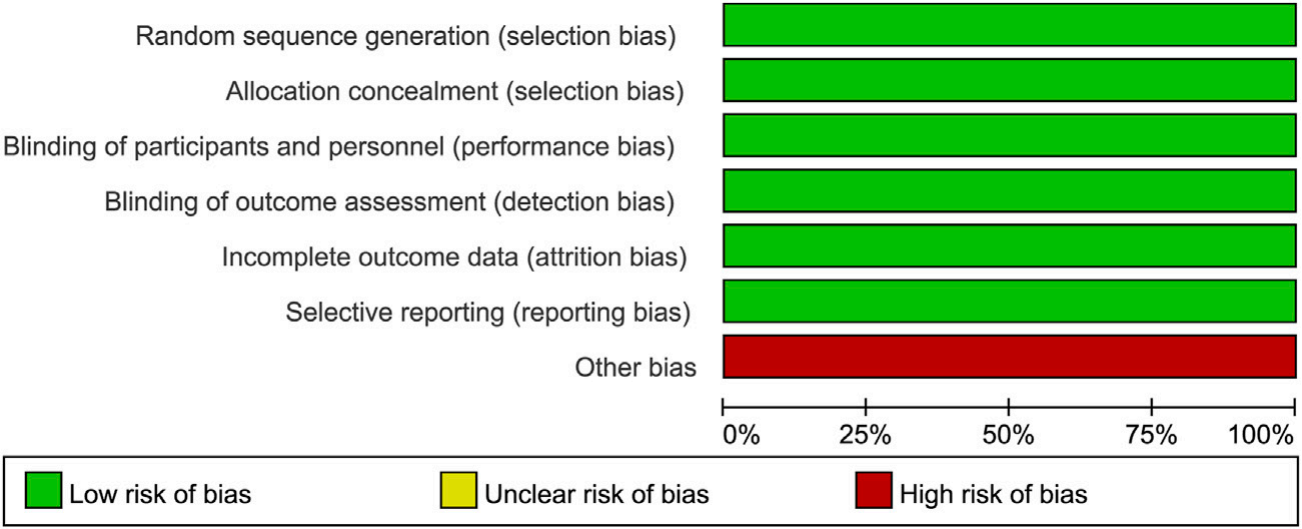
Risk of bias graph.

All studies correctly implemented the generation of a random sequence, participant and personnel blinding, concealment of allocation, and blinding of outcome assessment. None of the data were selectively reported in any of the studies. However, all four RCTs included in our study was funded by the same company that produced the drug (finerenone), which may have led to financial conflicts of interest (Figures 2, 3). Overall, there was a low-to-moderate risk of bias in the included trials.

### 3.4 Sensitivity analysis and publication bias

To evaluate the robustness of the results, a sensitivity analysis was carried out using a variety of statistical techniques, such as the Mantel-Haenszel random-effect and Peto fixed-effect models. The results showed no change in the direction of the effect, suggesting that the results were robust. Owing to the relatively short number of included trials, we employed Egger’s test via STATA software to assess publication bias. We found no publication bias results (*p* > 0.05).

### 3.5 Main outcomes

#### 3.5.1 Overall neoplasms

**FIGURE 4:**
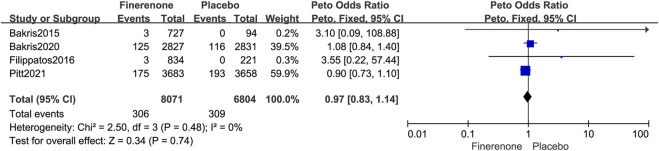
Forest plot of overall neoplasms based on finerenone vs. placebo.

**FIGURE 5:**
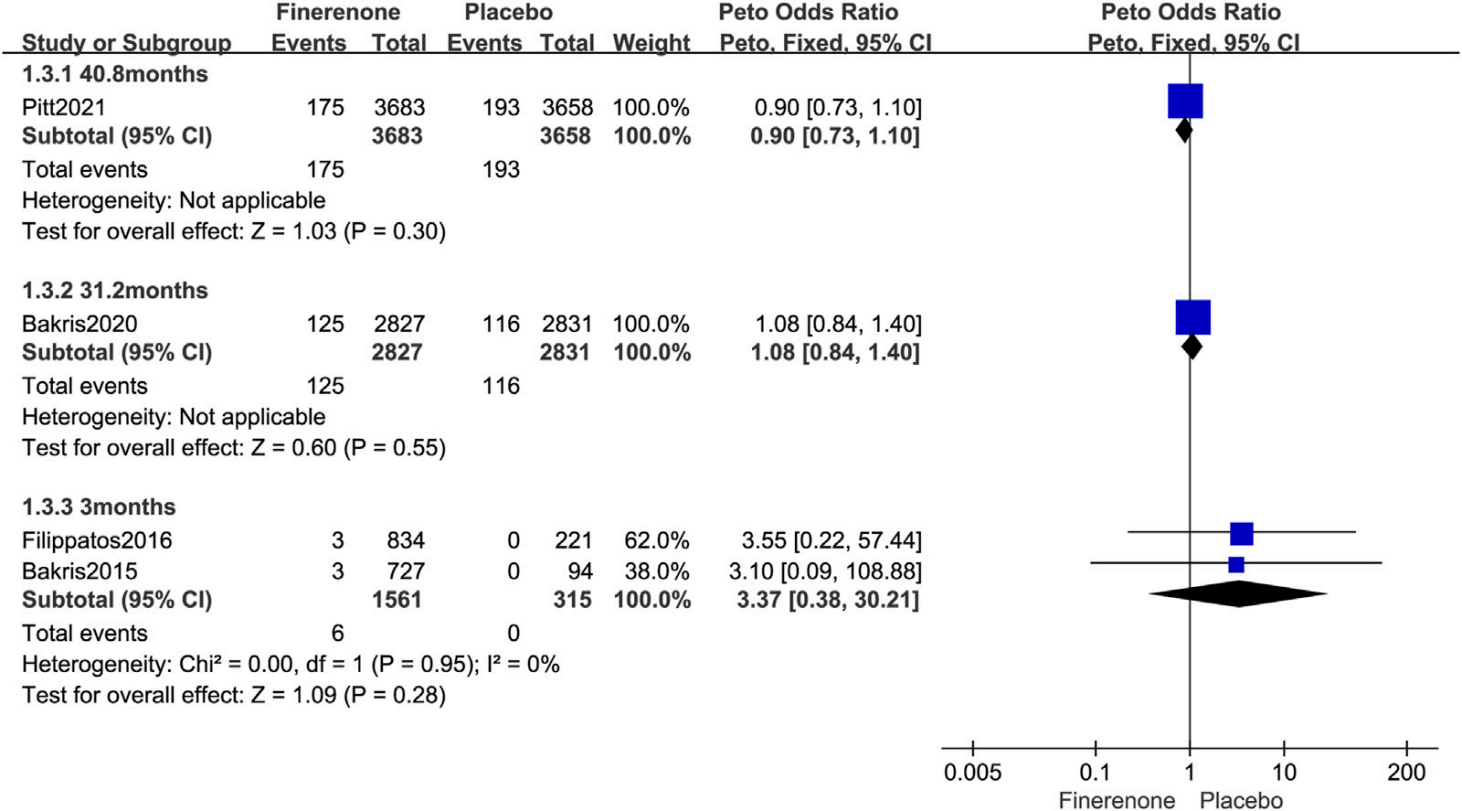
Forest plot of overall neoplasms at different follow-up times based on finerenone vs. placebo.

Four published RCTs reported the overall tumor risks of finerenone in patients with T2DM complicated by CKD and enrolled 14,875 patients with 615 tumors. Compared to placebo treatment, finerenone was not associated with an increased overall tumor risk (Peto OR = 0.97; 95% CI, 0.83–1.14; I^2^ = 0; 306/8,071 cases of cancer among patients treated with finerenone vs. 309/6,804 cases of cancer among those treated with placebo; Figure 4). The longer period of finerenone use did not increase neoplasm risk either (3 months: Peto OR = 3.37; 95% CI, 0.38–30.21; 6/1,561 vs. 0/315; 31.2 months: Peto OR = 1.08; 95% CI, 0.84–1.40; 125/2,827 vs. 116/2,831; 40.8 months: Peto OR = 0.90; 95% CI, 0.73–1.10; 175/3,683 vs. 193/3,658; Figure 5).

#### 3.5.2 Malignant neoplasms

**FIGURE 6:**
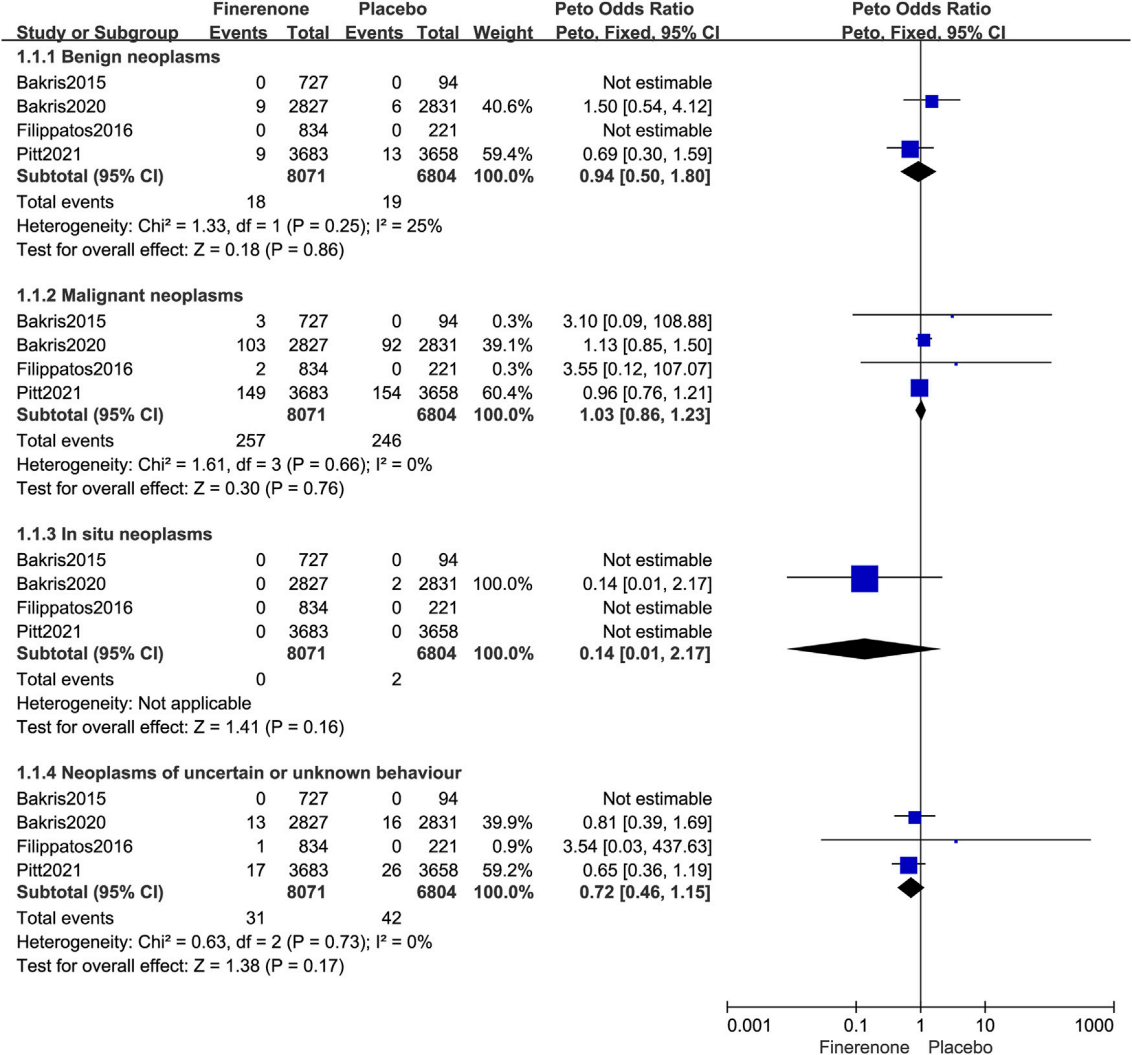
Forest plot of subgroup analysis by tumor nature based on finerenone vs. placebo.

Four published RCTs involving 14,875 patients with T2DM complicated by CKD reported 503 malignancies (Peto OR = 1.03; 95% CI, 0.86–1.23, I^2^ = 0%; 257/8,071 vs. 246/6,804; Figure 6). Four RCTs reported 13 systems for malignant risks. Finerenone was associated with an increased risk of malignant neoplasms of urinary tract when compared to placebo (Peto OR = 1.69; 95% CI, 1.07–2.67; 47/8,071 vs. 27/6,804; Table 2). Subgroup analysis was performed according to the organ source of the malignancy, there was no statistically significant difference in the risk of malignant neoplasms of the urinary organs between finerenone and placebo (kidney, except renal pelvis: Peto OR = 1.54; 95% CI, 0.68–3.49; 14/8,071 vs. 9/6,804; bladder: Peto OR = 1.72; 95% CI, 0.93–3.16; 27/8,071 vs. 15/6,804; other and unspecified urinary organs: Peto OR = 1.94; 95% CI, 0.52–7.17; 6/8,071 vs. 3/6,804; Figure 8).

**TABLE 2:** Malignant neoplasms subgroup analyzed by system.

Malignant neoplasms	Finerenone		Placebo		Peto OR (95% CI)
	No. of cancer	No. of participants	No. of cancer	No. of participants	
Lips, oral cavity and pharynx	10	8,071	4	6,804	2.35 [0.83, 6.71]
Digestive organs	69	8,071	71	6,804	0.96 [0.69, 1.34]
Respiratory and intrathorcic organs	33	8,071	30	6,804	1.10 [0.67, 1.80]
Bone and articular cartilage	0	8,071	2	6,804	0.13 [0.67, 1.80]
Melanoma and other malignant neoplasms of skin	11	8,071	10	6,804	1.10 [0.47, 2.59]
Mesothelial and soft tissue	4	8,071	1	6,804	3.32 [0.58, 19.18]
Breast	9	8,071	12	6,804	0.75 [0.32, 1.76]
Female genital organs	6	8,071	5	6,804	1.19 [0.37, 3.89]
Male genital organs	29	8,071	38	6,804	0.72 [0.44, 1.17]
Urinary tract	47	8,071	27	6,804	**1.69 [1.07, 2.67]^#^**
Eyes, brain and other parts of central nervous system	0	8,071	0	6,804	Not estimable
Thyroid and other endocrine glands	2	8,071	5	6,804	0.42 [0.10, 1.86]
Ill-defined,secondary and unspecified sites	26	8,071	29	6,804	0.87 [0.51, 1.49]
Lymphoid, haematopoietic and related tissue	11	8,071	12	6,804	0.91 [0.40, 2.07]

^#^Bold means statistically significant.

**FIGURE 7:**
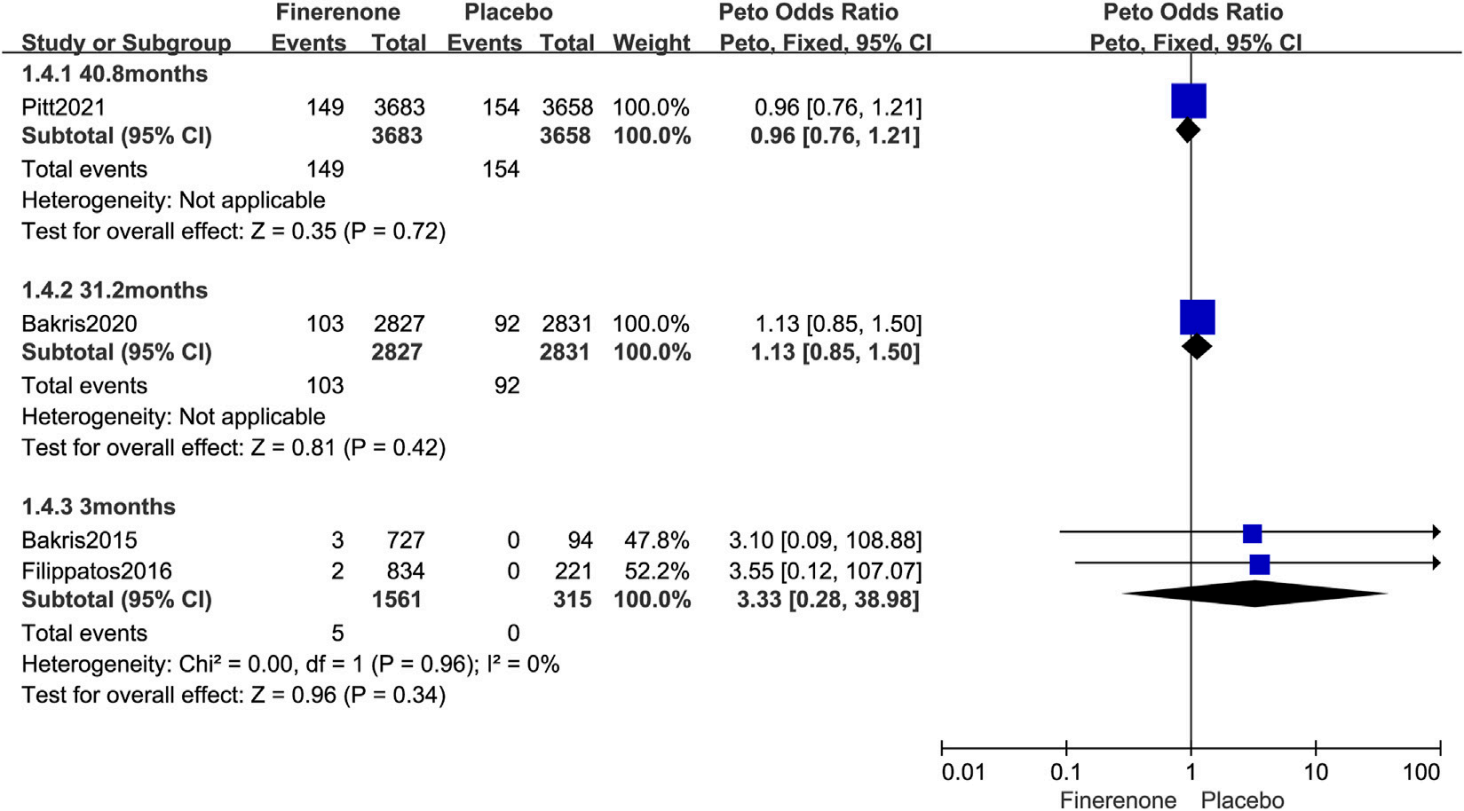
Forest plot of malignant neoplasms at different follow-up times based on finerenone vs. placebo.

Further subgroup analysis was performed based on the follow-up time for malignant neoplasms. The usage of finerenone for a longer period of time did not increase the risk of malignant neoplasm (3 months: Peto OR = 3.33; 95% CI, 0.28–38.98; 5/1,561 vs. 0/315; 31.2 months: Peto OR = 1.13; 95% CI, 0.85–1.50; 103/2,827 vs. 92/2,831; 40.8 months: Peto OR = 0.96; 95% CI, 0.76–1.21; 149/3,683 vs. 154/3,658; Figure 7).

#### 3.5.3 Benign neoplasms

Two studies (Bakris et al., 2020; Pitt et al., 2021) reported the occurrence of benign neoplasms. Compared with placebo, treatment with finerenone was not correlated with a higher incidence of benign neoplasms (Peto OR = 0.94; 95% CI, 0.50–1.80; I^2^ = 25%; 18/8,071 vs. 19/6,804; Figure 6).

#### 3.5.4 Neoplasms of uncertain or unknown behavior

Three studies (Filippatos et al., 2016; Bakris et al., 2020; Pitt et al., 2021) reported the occurrence of neoplasms with undetermined or unknown dynamics. Compared with placebo, finerenone was not correlated with higher tumor risks of uncertain or unknown behavior (Peto OR = 0.72; 95% CI, 0.46–1.15, I^2^ = 0%; 31/8,071 vs. 42/6,804; Figure 6).

#### 3.5.5 *In situ* neoplasms

Only one study (Bakris et al., 2020) reported the occurrence of neoplasms *in situ*, and the risks of *in situ* neoplasms were not higher with finerenone than with placebo (Peto OR = 0.14; 95% CI, 0.01–2.17; 0/8,071 vs. 2/6,804; Figure 6).

## 4 Discussion

### 4.1 Main findings

**FIGURE 8:**
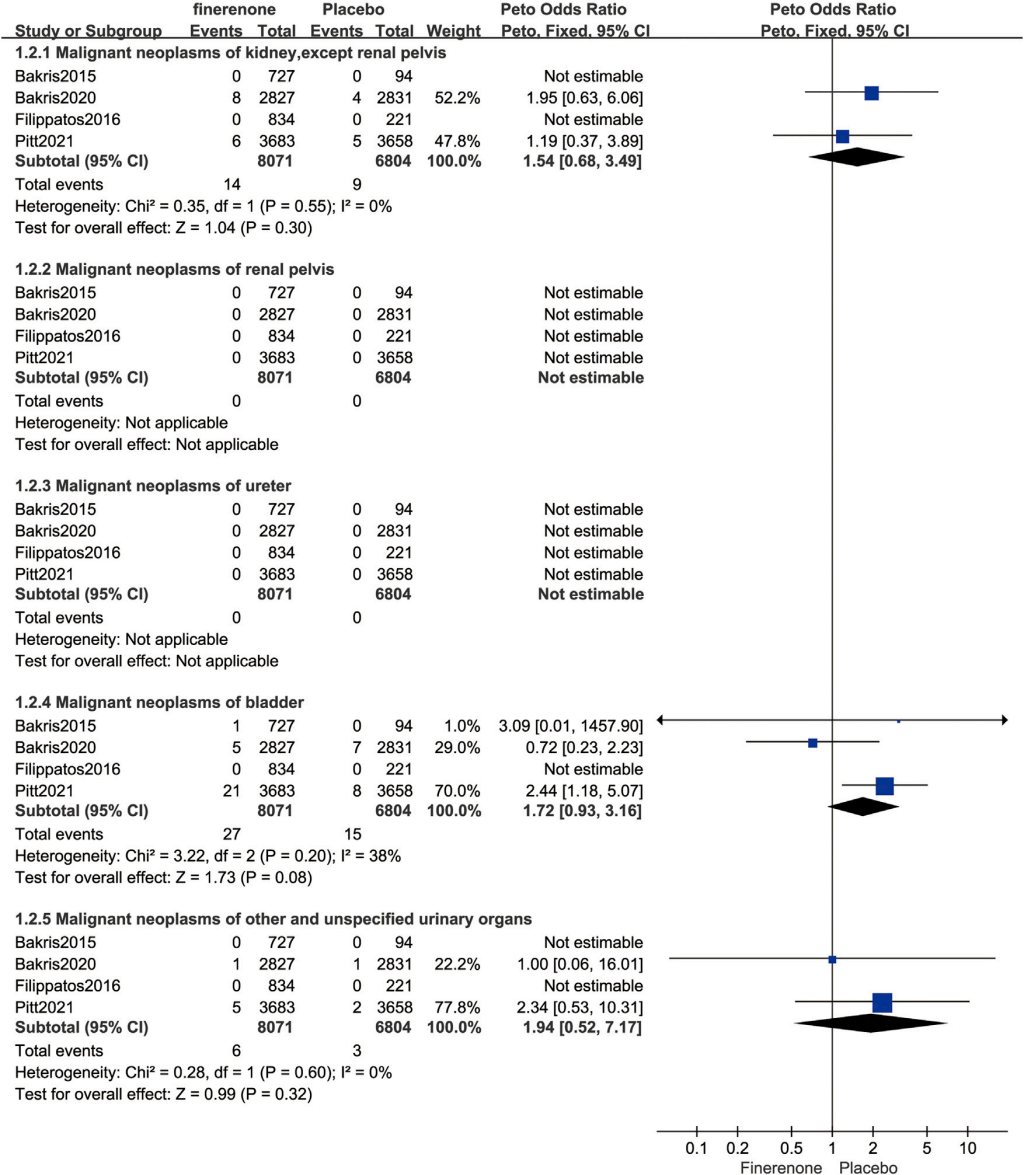
Forest plot of malignant neoplasms of urinary organ based on finerenone vs. placebo.

Our investigation is, as far as we are aware, the first systematic review and meta-analysis to evaluate the tumor risks of finerenone in patients with T2DM exacerbated by CKD. When compared to placebo, finerenone was not associated with increased risks of overall tumor (Peto OR = 0.97; 95% CI, 0.83–1.14), malignant neoplasms (Peto OR = 1.03; 95% CI, 0.86–1.23), benign neoplasms (Peto OR = 0.94; 95% CI, 0.50–1.80), *in situ* neoplasms (Peto OR = 0.14; 95% CI, 0.01–2.17), or neoplasms of uncertain or unknown behavior compared with placebo treatment (Peto OR = 0.72; 95% CI, 0.46–1.15). Moreover, finerenone had an increased risk of malignant neoplasms of urinary tract (Peto OR = 1.69; 95% CI, 1.07–2.67). Subgroup analysis was conducted based to the organ source of the malignancy. The results showed no statistically significant difference in the risk of malignant neoplasms of the urinary organs between finerenone and placebo. However, it was observed that finerenone increased the risk of malignant neoplasms of the bladder in certain RCTs (Peto OR = 2.44; 95% CI, 1.18–5.07; Figure 8) (Pitt et al., 2021). Further statistical analysis of the data revealed that while individual trial data suggested an increased risk of malignant neoplasms of the bladder with finerenone, the combined data did not show a significant increase in risk, taking into account the limited sample size included.

In people who have arterial hypertension, heart failure, reduced ejection fraction, and CKD, MR blocking has been shown to have definite therapeutic efficacy (Bauersachs et al., 2015). The use of steroid mineralocorticoid receptor antagonists (MRAs) SPIR and eplerenone has been limited due to the risk of hyperkalemia and renal impairment (Svensson et al., 2004; Dinsdale et al., 2005; Vukadinović et al., 2017). Accordingly, scientists have developed a new nonsteroidal MRAs finerenone (Bärfacker et al., 2012), which has higher receptor selectivity (Bärfacker et al., 2012) and a lower risk of hyperkalemia (Pitt et al., 2012) and renal protective effects (Bakris et al., 2015; Bakris et al., 2020), and has been approved to be released in the market in 2021 (Frampton, 2021).

According to published research, SPIR is also associated with a reduced risk of prostate cancer (Mackenzie et al., 2017; Hiebert et al., 2021; Bommareddy et al., 2022). Leung et al. (2013) showed that SPIR inhibited the metastasis of colon cancer cells. Specific mechanisms include reducing the content of cancer stem cells, inhibiting the repair frequency of cancer DNA, and enhancing the sensitivity of cancer cells to chemotherapy (Alekseev et al., 2014; Shahar et al., 2014; Gold et al., 2019). Another MAR, eplerenone, affects the occurrence and development of hepatocellular carcinoma by inhibiting angiogenesis and expression of vascular endothelial growth factor and promoting the apoptosis of cancer cells (Kaji et al., 2010).

Previous studies have shown that naturally derived naphthyridines have anti-infective, anti-cancer, and immune-regulatory effects (Chabowska et al., 2021). The biosynthesis of naphthyridine derivatives also has anti-cancer, anti-microbial, anti-inflammatory, anti-oxidation, immune-regulation, and other effects (Madaan et al., 2015; Lavanya et al., 2021). Finerenone and its metabolites are naphthyridine derivatives (Gerisch et al., 2018; Chabowska et al., 2021). Our research revealed that finerenone was associated with an increased risk of malignant neoplasms of the urinary tract. Our findings are inconsistent with previous studies of the pharmacological effects of naphthyridines derivatives. Further research is needed to explore whether the increased risk of malignant neoplasms of the urinary tract caused by finerenone is related to its excretion pathway or whether finerenone and its metabolites have other pharmacological effects that we have not yet identified.

Kitchlu et al. (2022) found an increased risk of cancer in patients with CKD. Similarly, Kurasawa et al. (2023) reported a U-shaped relationship between estimated glomerular filtration rate and cancer incidence. The FDA has previously warned about the potential risk of medullary thyroid cancer with glucagon-like peptide-1 receptor agonists. García et al. (2021) analyzed cases from the European Pharmacovigilance Database and discovered a higher number of bladder cancer cases among users of sodium-glucose cotransporter-2 inhibitors. Yarmolinsky et al. (2022) studied the cancer risk associated with angiotensin-converting enzyme inhibitors, β-blockers, and thiazide diuretics, finding that long-term use of ACE inhibitors was linked to an increased risk of colorectal cancer. Dąbrowski reported that insulin had a dose-dependent cancer risk, while metformin could reduce cancer incidence and indirectly inhibit tumor growth (Dąbrowski, 2021). Our study focused on patients with T2DM and CKD who received concurrent treatment with antidiabetic, antihypertensive, and diuretic medications. Due to limitations in the trial data, we did not explore variations in tumor risk based on CKD stage or evaluate the impact of concomitant therapy on tumorigenesis.

### 4.2 Limitations

This systematic review and meta-analysis had certain limitations. The studies analyzed were limited to RCTs, with no cohort or case-control studies involved. While RCTs can address baseline measures, reduce bias, and minimize confounding factors, some studies had small sample sizes and brief follow-up periods, with the minimum follow-up being only 3 months. For rare occurrences like tumors, small sample sizes may not be sufficient to detect meaningful results. Additionally, the number of studies analyzed was limited, with only four RCTs meeting the criteria for inclusion. None of the included studies examined population-specific cancer differences between men and women.

## 5 Conclusion

Our findings suggest that finerenone is not correlated with an increased risk of overall tumor development in patients with T2DM complicated with CKD, whereas subgroup analysis by tumor system suggested that finerenone might promote the risk of malignant neoplasms of urinary tract. Nevertheless, due to the small sample size and relatively short follow-up period, further well-planned and larger study populations are warranted.

## Data Availability

The original contributions presented in the study are included in the article/Supplementary material, further inquiries can be directed to the corresponding authors.
